# Negative immune factors might predominate local tumor immune status and promote carcinogenesis in cervical carcinoma

**DOI:** 10.1186/s12985-016-0670-8

**Published:** 2017-01-13

**Authors:** Minyi Zhao, Yang Li, Xing Wei, Qian Zhang, Hongran Jia, Shimin Quan, Di Cao, Li Wang, Ting Yang, Juan Zhao, Meili Pei, Sijuan Tian, Yang Yu, Yanping Guo, Xiaofeng Yang

**Affiliations:** 1 Department of Obstetrics and Gynecology, First Affiliated Hospital of Xi’an Jiaotong University, 277 West Yanta Road, Xi’an, 710061 China; 2 Department of Obstetrics and Gynecology, First Affiliated Hospital of Xi’an Jiaotong University, Xi’an, China

**Keywords:** Cervical cancer, Immune microenvironment, Immune factors

## Abstract

**Background:**

The disequilibrium of local immune microenvironment is an essential element during tumorigenesis.

**Method:**

By conducting real-time polymerase chain reaction, we identified the mRNA level of immune factors, FoxP3 (forkhead box protein P3), CCL22/CCR4 (chemokine (C-C motif) ligand 22/CC chemokine receptor 4), OX40L/OX40 (tumor necrosis factor superfamily member 4/tumor necrosis factor receptor superfamily member 4) and Smad3 (SMAD family member 3) in neoplastic foci and its periphery tissues from 30 cases of squamous cervical carcinoma and 20 cases of normal cervix.

**Result:**

The FoxP3, CCL22 and CCR4 mRNA level in local immune microenvironment of normal cervix was lower than that in cervical cancer. While OX40L, OX40 and Smad3 mRNA level profile in normal cervix was higher than that in cervical cancer. Beyond individual effect, the pairwise positive correlations were demonstrated among the mRNA level of FoxP3, CCL22 and CCR4. The mRNA level of OX40 negatively correlated with CCL22, but positively correlated with Smad3. Moreover, the mRNA level of FoxP3 and CCL22 was increased while Smad3 was decreased in cervical tissue with HPV (human papilloma virus) infection.

**Conclusion:**

Our data yields insight into the roles of these immune factors in cervical carcinogenesis. It may therefore be that, in microenvironment of cervical squamous cell carcinoma, along with the context of HPV infection, negative immune regulators FoxP3, CCL22 and CCR4 might overwhelm positive immune factors OX40L, OX40 and Smad3, giving rise to an immunosuppressive status and promote the progression of cervical carcinogenesis.

**Trial registration:**

Not applicable.

## Background

Cervical cancer is the fourth most common female malignant disease globally, posing a serious threat to women’s health. The latest data released by WHO showed that there were 528,000 new cases and 266,000 deaths all over the world in 2012. Roughly 62,000 new cases were reported in China along with 30,000 deaths [1]. Worse still, cervical cancer is becoming more and more prevalent among young Chinese women [2]. Epidemiological evidence has confirmed that HR-HPV (high-risk human papilloma virus) is the trigger of cervical cancer. Normally, HPV infection is frequent among women and about 90% can be spontaneously cleared [3–5]. While 10% persists, which is crucial to malignant progression. Studies have illustrated that HPV exploits kinds of mechanisms to hide from being inactivated, such as restraining the activity of NK (natural killer cells) [6–8] and LCs (langerhans cells) [9]. Tumor immune microenvironment is a complex milieu maintaining a delicate balance between inflammation and tolerance [10]. Once broken, host cells infected by HR-HPV can’t be eliminated effectively, allowing for tumorigenesis, tumor progression and metastasis.

FoxP3 + Treg has been identified as a notorious immunosuppressive cell in local site. FoxP3 is regarded as a critical marker to the development and negative immune effect of FoxP3 + Tregs. CCL22 is one of CC chemokines produced by macrophages, dendric cells and tumor cells. CCR4, confirmed as its specific receptor, is mainly expressed in different T cell subsets, including Th2 (helper T cell) and regulatory T cells. The CCL22-CCR4 axis could induce the expression of FoxP3 in Tregs [11] and infiltrate FoxP3 + Treg selectively into tumor sites, contributing to form an immunocompromised environment in favor of tumor growth. OX40 is highly expressed on effector T cells and Tregs. Its ligand named OX40L, is mostly localized on activated APC (antigen presenting cell). Plenty of evidences have demonstrated that OX40L-OX40 axis is of great importance to impede the suppressive function of Treg by inhibiting TGF-β-driven (transforming growth factor-β) conversion of naive CD4^+^ FOXP3^−^ T cells into CD4^+^FOXP3^+^ T cells [12]. Smad3 is a pivotal intracellular mediator for participating in the activation of multiple immune signal pathway. TNF-α (tumor necrosis factor-α) impairs the differentiation and function of TGF-β-induced FoxP3 + Tregs by inhibiting the phosphorylation of Smad3. Moreover, studies from Smad^−/−^ mice have testified that the loss of Smad3 induced IgG2a expression, leading to an obvious enhanced Th2 reaction and Th1/Th2 shift [13]. Low expression of Smad3 has been found in acute T-cell lymphoblastic leukemia [14] and gastric carcinoma [15].

Hitherto, the expression profile of these immune factors in the immune microenvironment of cervical cancer has not yet been identified. We therefore examined their expression in cervical tissues with the aim of delineating their individual and underlying combined effects in modulating the local immune state and tumorigenesis.

## Methods

Tissues samples from 30 patients with cervical squamous cell carcinoma and 20 patients with hysteromyoma treated in the department of gynecology of The First Affiliated Hospital of Xi’an Jiaotong University were recruited from 2014 to 2015, with an median age of 43.6 (range 27–62) years. All the cancer cases were diagnosed without lymphovascular invasion or lymphatic metastasis, including 22 cases of low grade squamous cell carcinoma and 8 cases of high grade squamous cell carcinoma, with 27 HPV-positive cases and 3 HPV-negative cases. The control group comprised 6 HPV-positive cases and 14 HPV-negative cases.

### Sample collection

After the uterus was removed in the surgery, we collected about 2x1x1cm^3^ normal cervix or neoplastic foci and pericarcinous tissues of the same size on or beside the cervical lesions macroscopically and rinsed the blood out of the specimen with PBS buffer in sterile condition immediately. These specimens were preserved in liquid nitrogen or −80 °C refrigerator.

### Real-time PCR

Total RNA was extracted and purified from tissue specimen using TRIzol Reagent (15596-026, life, USA). The RNA concentration was determined by spectrophotometric assay.4 ug of total RNA with 0.4uM of specific primer was then subjected into cDNA using PrimeScript^TM^ RT Master Mix (RR037A, TaKaRa Japan), followed by real-time PCR using SYBR Prime Ex Taq^TMII^ (RR820A, TaKaRa Japan) according to the manufacturer’s instruction (C1000 Thermal Cycler, BIO-RAD, USA). The primers used were as follows (Table 1). The expression level of targeted genes was calculated using internal control GAPDH mRNA and the 2^-△△Ct^ methods.

**Table 1:** The primers for each immune factors and house-keeping gene

Gene	Primer sequence	Product of amplication (bp)
FoxP3-F	TTTGCTGATTGTTGCTTTGC	
FoxP3-R	GTTCAACTGATGCTGCCTGA	158
CCL22-F	GGTATTTGAACCTGTGGAATTGGAG	
CCL22-R	CAGGCCCTGGATGACACTGA	163
CCR4-F	CCCTTAGGGATCATGCTGTT	
CCR4-R	TCAAAGGTGCAGTCCTGAAG	197
OX40-F	CAAGCCTGGAGTTGACTGTG	
OX40-R	GATTGCGTCCGAGCTATTG	139
Smad3-F	AGACCTCATGCCCAGCTCTC	
Smad3-R	GGGGAGGGAGACAGACAAAAC	165
OX40L-F	TCCCAGCCTCCAAACATAGT	
OX40L-R	GTGTTGCTTTGCCTGTCTGT	359
GAPDH-F	GCACCGTCAAGGCTGAGAAC	
GAPDH-R	TGGTGAAGACGCCAGTGGA	138

### Statistical analysis

All data was processed by SPSS 20.0 statistical software. Mann-Whitney U rank sum test and Spearman rank correlation analysis were chosen for data managing. A *P* value of less than 0.05 was considered to indicate statistical significance.

## Results

1. Negative immune regulators, FoxP3, CCL22 and CCR4, prevailed in cervical cancer immune microenvironment.
  To ascertain FoxP3, CCL22 and CCR4 mRNA level in cervical carcinoma, we quantified their expression in 30 cases of neoplastic foci with adjacent tissue and 20 healthy control. The results exhibited the mRNA level of FoxP3, CCL22 and CCR4 of local microenvironment in cervical cancer was significantly higher than that in normal cervix. Besides, we also compared their mRNA level in different pathological grade . In contrast to low grade squamous cell carcinoma, their mRNA level increased moderately in high grade lesion (Fig. 1).

**Fig. 1:**
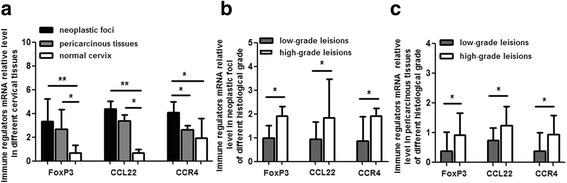
FoxP3, CCL22 and CCR4 mRNA relative level in cervical tissues. Figure **a** shows significantly higher level of FoxP3, CCL22 and CCR4 mRNA in neoplastic foci and (or) periphery tissues than that in normal cervix. Figure **b** shows higher level of FoxP3, CCL22 and CCR4 mRNA in neoplastic foci of high grade squamous cell carcinoma than that in low grade lesion. Figure **c** reveals higher level of FoxP3, CCL22 and CCR4 mRNA in pericarcinous tissues of high grade squamous cell carcinoma than that in low grade lesion. *P < 0.05, **P < 0.01
2. Positive immune factors, OX40L, OX40 and Smad3, crippled in cervical cancer immune microenvironment.
  The positive immune enhancers, OX40L, OX40 and Smad3 mRNA level of local microenvironment in cervical cancer was significantly lower than that in normal cervix. In comparison to low grade squamous cell carcinoma, their mRNA level decreased moderately in high grade lesion (Fig. 2).

**Fig. 2:**
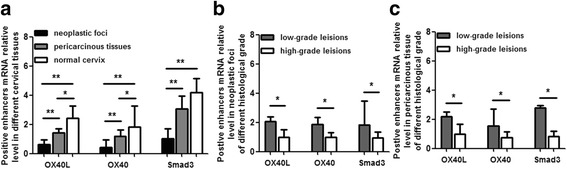
OX40L/OX40 and Smad3 mRNA relative level in cervical tissues. Figure **a** shows significantly higher level of OX40L/OX40 and Smad3 mRNA in normal cervix than that in neoplastic foci and (or) periphery tissues. Figure **b** shows lower level of OX40L/OX40 and Smad3 mRNA in neoplastic foci of high grade squamous cell carcinoma than that in low grade lesion. Figure **c** reveals lower level of OX40L/OX40 and Smad3 mRNA in pericarcinous tissues of high grade squamous cell carcinoma than that in low grade lesion. *P < 0.05, **P < 0.01
3. The synergism of congeneric immune factors and antagonism of alien regulators might worsen the immune imbalance in cervical cancer microenvironment.
  To discover whether mutual effect existed in local site among these factors, correlation analysis was conducted of their mRNA level. The results showed that the mRNA level of CCL22 was positively correlated with FoxP3 in neoplastic foci and adjacent tissues (*r* = 0.353, *r* = 0.307, *P* < 0.05) . Parallel outcome was observed between the mRNA level of FoxP3 and CCR4 (*r* = 0.607, *r* = 0.205, *P* < 0.05), CCL22 and CCR4 (*r* = 0.786, *r* = 0.344, *P* < 0.05), as well as OX40 and Smad3 (*r* = 0.384, *r* = 0.288, *P* < 0.05). The correlation analysis indicated that in immune microenvironment of cervical cancer, the mRNA level of CCL22 and CCR4 was strengthened as the Foxp3 mRNA level increased and vice verse. The pairwise correlation prompted that inhibitory regulators, FoxP3, CCL22 and CCR4 may magnify each other’s negative function through inducing mutual expression of mRNA, then synergistically promote local immunosuppression. As the mRNA level of OX40 and Smad3 was decreased in neoplastic foci and pericarcinous tissues, the potential congenerous effect of OX40 and Smad3 might be weakened in local microenvironment of cervical cancer. Conversely, the mRNA level of CCL22 seemed to be inversely related to OX40 (*r* = −0.288, *r* = −0.263, *P* < 0.05), which hinted us that there still underlies counteraction between CCL22 and OX40 to accentuate immune inhibition. While no correlations were found between other factors
4. Immune repressors, FoxP3 and CCL22, were vibrant while promoter Smad3 was sluggish in HPV-positive local cervical microenvironment.
  To assess the association between HPV infection and immune factors mRNA level, the total 50 samples were divided into HPV-positive and HPV-negative group and comparison was performed. The results displayed higher level of mRNA of FoxP3 and CCL22 in HPV-positive cancerous and adjacent tissues comparing to HPV-negative corresponding tissues. While lower level of Smad3 mRNA was observed in HPV-positive cancerous tissues than that in HPV-negative cases. All these outcomes indicated that in the backdrop of HPV infection, the immunosuppressive impact of immune repressors might be aggravated while immunological acceleration of enhancer be passivated which tilted the immune status to the tolerance condition. The mRNA level of CCR4, OX40L and OX40 were invariable in cervical tissues regardless of different HPV infection status (Fig. 3).

**Fig. 3:**
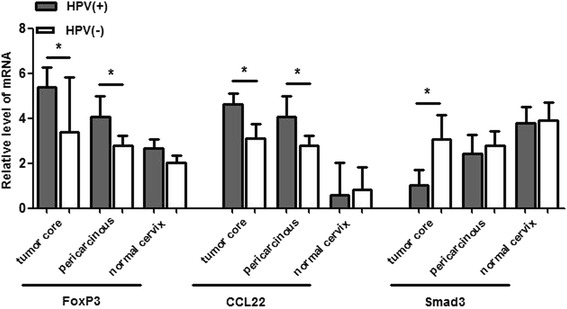
The mRNA level of immune factors in different HPV infection status. FoxP3 and CCL22 mRNA level was up-regulated, while Smad3 mRNA level was down-regulated in HPV-positive cases compared to the HPV-negative corresponding group. *P < 0.05

## Discussion

1. Negative immune factors might play an dominant role in suppressing local tumor immune status and promoting carcinogenesis in HPV triggered cervical cancer.
  As a momentous immunosuppressive cell, FoxP3 + Tregs is suspected of reducing T cell activity [16] and secreting repressive cytokines, such as interleukin-10 [17] and interleukin-35. Intriguingly, although more and more data had provided first evidence for FoxP3 expression localized also in cancer cells, our preliminary trial has showed that the positive expression of FoxP3 was only observed in the nucleus of regulatory T cells rather than in cervical carcinoma cell lines or cervical cancer cells by immunohistochemical staining.
  In comparison to normal cervix, the mRNA level of these negative factors exhibited a remarkable increase in microenvironment of cervical cancer. Moreover, these factors expressed significantly more in high grade squamous carcinoma cell than in low grade lesion. Our data presented here implied that FoxP3 and CCL22/CCR4 played an vital part in down-regulating immune response of tumor microenvironment and boosting the cervical cancer carcinogenesis. Meanwhile, the less differentiated the cervical cancer cell was, the higher the mRNA level of FoxP3, CCL22 and CCR4 would be in microenvironment of cervical cancer, exacerbating the disequilibrium of local immunity and tumor growth. It was in coincidence with our prophase study which showed that the number of FoxP3 + Tregs was lowest in normal cervix and highest in invasive neoplasia and compared to high grade squamous cell carcinoma, it diminished in low grade lesions. Kim et al. [18] observed that the distribution frequency of FoxP3 + Treg was extremely high in colorectal, pancreatic, hepatocelluar and ovarian carcinoma [19–21] and Kumai et al. noticed high expression of CCL22 and CCR4 in nasal NK/T cells lymphoma, lung and breast cancer [22–24].
  As mentioned above, HPV infection is the key causative element of cervical carcinogenesis. Several studies have found that the expression product of HPV can convert local cytokines profiles in cervical lesion called Th1/Th2 shift by decreasing the levels of Th1 cytokines and increasing the Th2 cytokines [25]. In our research, we noticed higher level of FoxP3mRNA and CCL22mRNA in cancerous and adjacent tissues with HPV infection, which reminded us that in the context of HPV infection, immune regulators that favor negative effect could be accumulated in local situs to incline the immune status towards suppressive side.
2. Inspiring anti-tumor regulators were overwhelmed in cervical cancer immune microenvironment and conducive to carcinogenesis.
  Compared to normal cervix, the mRNA level of OX40L/OX40 and Smad3 displayed a decrease in cervical cancer microenvironment. These factors expressed less in high grade squamous carcinoma cell than in low grade lesion, speculating that in immune microenvironment of cervical cancer, the positive role of OX40L/OX40 and Smad3 in up-regulating immune response and repressing the cervical carcinogenesis was impaired. In addition, the less differentiated the cervical cancer cell was, the weaker these enhancers would be in anti-tumor immunological effect, intensifying the unbalance of local immunity and tumorgenesis.
  In cancerous tissues, the level of Smad3mRNA with HPV infection is lower than that in HPV-negative group, cluing us HPV could discourage the distribution of enhancer in local situs towards suppressive side.
3. The underlying synergistical effect between congeneric immune factors and the counteraction between alien regulators may aggravate the immune suppression in cervical cancer microenvironment.
  The positive correlation between mRNAs of FoxP3 and CCL22, FoxP3 and CCR4, CCL22 and CCR4 elucidated that FoxP3, CCL22 and CCR4 might synergistically promote immunosuppression. We surmise that one one hand, local overexpression of CCL22 and CCR4 strengthened the differentiation and aggregation of FoxP3 + Treg in the tumor site, thus inhibiting anti-tumor immunity and maintaining a hostile environment . On the other hand, immunosuppressive cytokine IL-4 (interleukin-4) and IL-1β (interleukin-1β) secreted by FoxP3 + Treg could induce the excretion of CCL22 from macrophages, dendric and tumor cells. Meanwhile, FoxP3 could promote CCR4 expression in the same Treg cell. Our results, OX40mRNA positively correlated with Smad3mRNA, but negatively correlated with CCL22mRNA implied that for one thing, as the mRNA level of OX40 and Smad3 were decreased conformably in neoplastic foci and pericarcinous tissues in comparison to normal cervix, the potential cooperative effect of these two factors might be weakened in local microenvironment of cervical cancer. For another thing, the effective immune response of OX40 could be impaired by CCL22 as the high mRNA level of CCL22 in local microenvironment seemed to be inversely related to OX40. No significant relationship was explored between other factors, prompting that there is no direct interaction involved between them in the regulation of immune state. For instance, we did not find any relationship between FoxP3 mRNA and Smad3mRNA.As previously mentioned, phosphorylation of Smad3 is the functional variant in pathways which mediated FoxP3 transcription. It hinted us that whether the lower level of Smad3 mRNA in local cancer microenvironment may leads to the decrease of the ratio of p-Smad3 and total Smad3, thereby repressing the FoxP3 mRNA level.

## Conclusions

In summary, these results showed that the level pattern of mRNA of FoxP3, CCL22/CCR4, OX40L/OX40 and Smad3 in cervical cancer immune microenvironment distinctly differed from that in normal cervix. Since mRNAs of positive immune factors OX40L/OX40 and Smad3 decreased while negative immune factors FoxP3 and CCL22/CCR4 increased, we infer that negative immune regulation factors conquer and hold the leadership in local immunocompromised microenvironment. Besides, their latent complex relationship not only amplify immune tolerance, but also hinder anti-tumor immune response, which further gives rise to the break of equilibrium of local immunity. Such immune model may involve in the outcome of HPV infection and cervical carcinogenesis. We look forward to subsequent research to uncover the hidden mechanism.

## Abbreviations

APC: Antigen presenting cell
CCL22: Chemokine (C-C motif) ligand 22
CCR4: CC chemokine receptor 4
FoxP3: Forkhead box protein P3
HR-HPV: High-risk human papilloma virus
IL-1β: Interleukin-1β
IL-4: Interleukin-4
LCs: Langerhans cells
NK: Natural killer cells
OX40: Tumor necrosis factor receptor superfamily member 4
OX40L: Tumor necrosis factor superfamily member 4
Smad3: SMAD family member 3
TGF-β: Transforming growth factor-β
Th: Helper T cells
TNF-α: Tumor necrosis factor-α
